# The Thermometric Bureau

**Published:** 1880-10

**Authors:** 


### THE THERMOMETRIC BUREAU.

[We copy the following from the circular of the Horological and Thermometric Bureaus of Yale College, which may interest some of our readers.—ED.]

Statistics show that several thousand thermometers of refined construction, and graduated on the stem to 0°.2 F. or thereabouts, are annually procured by the medical practitioners of our country alone for physiological researches and daily practice. The majority of these thermometers are newly made (within six months), and their verification depends on inferior (from the scientific standpoint) thermometers in the hands of individual makers. It is needless to say that the readings of such thermometers have little value in indicating the true temperature of a patient, or affording data in cases which the physician wishes to describe in print.

The makers of thermometers in our country have been, in general, content to use for their standards thermometers which have been compared at some foreign observatory, or with some more easily accessible instrument in which they place confidence, in the hands of a friendly neighbor. Thus it happens that many thousand American clinical thermometers have been sold, which do not depend upon a comparison with a recognized standard for their scale readings. The result has been that the American instruments have suffered in the estimation of scientific practitioners. This is not so much the fault of the American makers as their misfortune in not having the same facilities offered them by the properly equipped observatories this side of the water, which their favored competitors enjoy abroad.

The meteorological observers in this country have now no common standard of easy access; and it seems eminently proper that the observatory should undertake to be useful to the medical profession and the meteorologists in this country, and afford the means of comparison desired. With this end in view, the observatory has accepted the aid of the Board of Directors of the Bache Fund of the National Academy in obtaining the standards of the foreign observatories, and has made provision for the constant determination of the errors of the standards themselves. The following is the official circular of the Thermometric Bureau:

CIRCULAR CONCERNING THE VERIFICATION OF THERMOMETERS.

This Bureau has been established by the Corporation of Yale College, at the recommendation of the Board of Managers of the Winchester Observatory, in order to afford desired facilities for the adequate verification of thermometers.

Thermometers will be received by the observatory for the purpose of comparison with the observatory standards, and certificates of comparison signed by the astronomer in charge will be issued with thermometers so compared. These certificates will contain a statement of the corrections to be applied at intervals of five or ten degrees of the thermometer scale to cause it to have the same reading as the observatory standards. In general these corrections will be expressed in tenths of a degree Fahrenheit, or in twentieths of a degree Centigrade.

Thermometers sent for verification must have a name and number engraved upon them ; and thermometers which are not graduated on the glass stem must be of sufficiently good workmanship to satisfy the observer in charge that the scale will not suddenly change with reference to the glass stem of the thermometer tube, with ordinarily careful usage.

The Board of Managers have established the following scale of charges for this service, which includes the hall mark and the certificate :

| | |
|---|---|
| Standard Meteorological Thermometers | $1.00 |
| Ordinary Meteorological Thermometers | .50 |
| Ordinary Maximum Thermometers | .75 |
| Ordinary Minimum Thermometers | .75 |
| Clinical Thermometers | .50 |

There will be a deduction of one-fifth of the above charges where more than eight thermometers of one kind are received at the same time. In the case of clinical thermometers the charge will be four dollars per dozen when not less than two dozen are sent at the same time.

For other thermometers than the above, the charges for verification will be furnished on application.

The letter of advice accompanying thermometers sent for verification should contain the maker's name, the number of each thermometer, and full directions for reshipment.

All proper precautions are taken by the Board of Managers to guard against loss or injury ; but as it is manifestly inexpedient that a University corporation should be responsible for property in its care for such a purpose, it is to be understood that all risks are assumed by the person sending the thermometers.

LEONARD WALDO, *Astronomer in Charge.*

Approved and ordered to be published by the Board of Managers of the Winchester Observatory.

C. S. LYMAN, *President.* H. A. NEWTON, *Secretary.*

NEW HAVEN, CONN., June 1, 1880.

The observatory desires to encourage the general verification of thermometers on the part of the members of the medical profession, meteorological observers, and all those persons who have occasion to note temperatures to less than one degree Fahrenheit. The inaccuracies of the thermometers in use by the majority of such persons are considerably greater than is commonly supposed.

It will be seen that the observatory places every facility at the disposition of observers and thermometer makers for the ready verification of thermometers; and there is no good reason why a purchaser should not have an accurate knowledge of the errors of his instrument should he so desire.

The observatory will make arrangements with hospitals and other institutions using a number of thermometers, for the systematic examination at stated intervals of all thermometers in their use. Such an arrangement precludes errors arising from the use of newly made instruments which have been verified, but whose scales have not yet attained an approximately permanent position.

For the present the comparisons of clinical and meteorological thermometers will be made with a water bath in which the water is brought to a given temperature and mechanically agitated before the comparison is made. The standard to which the primary and secondary mercurial standards will be referred is the air thermometer.

Ordinary thermometers are returned within three days from the time of their reception, if the observatory charges for verification are remitted with the thermometers.

In case they are not so remitted, they are payable upon notification by the observatory that the thermometers are ready to be returned.

Dealers and manufacturers furnishing satisfactory references to the observatory may open an account, to be settled quarterly, beginning with January 1 of each year.

